# Code-VEP vs. Eye Tracking: A Comparison Study

**DOI:** 10.3390/brainsci8070130

**Published:** 2018-07-07

**Authors:** Hooman Nezamfar, Seyed Sadegh Mohseni Salehi, Matt Higger, Deniz Erdogmus

**Affiliations:** Cognitive Systems Laboratory, Electrical and Computer Engineering Department, Northeastern University, Boston, MA 02115, USA; ssalehi@ece.neu.edu (S.S.M.S.); higger@ece.neu.edu (M.H.)

**Keywords:** SSVEP, eye tracking, c-VEP, EEG, BCI

## Abstract

Even with state-of-the-art techniques there are individuals whose paralysis prevents them from communicating with others. Brain–Computer-Interfaces (BCI) aim to utilize brain waves to construct a voice for those whose needs remain unmet. In this paper we compare the efficacy of a BCI input signal, code-VEP via Electroencephalography, against eye gaze tracking, among the most popular modalities used. These results, on healthy individuals without paralysis, suggest that while eye tracking works well for some, it does not work well or at all for others; the latter group includes individuals with corrected vision or those who squint their eyes unintentionally while focusing on a task. It is also evident that the performance of the interface is more sensitive to head/body movements when eye tracking is used as the input modality, compared to using c-VEP. Sensitivity to head/body movement could be better in eye tracking systems which are tracking the head or mounted on the face and are designed specifically as assistive devices. The sample interface developed for this assessment has the same reaction time when driven with c-VEP or with eye tracking; approximately 0.5–1 second is needed to make a selection among the four options simultaneously presented. Factors, such as system reaction time and robustness play a crucial role in participant preferences.

## 1. Introduction

Assistive devices play an important role in the daily life of the individuals with disabilities. These devices can improve the quality of life by a great margin. However, the overall performance of these devices affects their usability. Factors such as, information transfer rate (ITR), error rate, reliability, robustness and supported applications are among the most considered factors determining how effective these devices are.

Delivering viable communication often requires customizing the interface to an individual’s unique abilities. Unfortunately, these considerations are among the most difficult to consider in the research setting where such devices are designed. This is true of the visual system, a popular input modality choice. The idea of interpreting the gaze position as the intended point has been used in many eye-tracking based interfaces. However, due to natural rapid saccade and fixation movements of the human eye, proper tracking requires complex real-time image processing, challenged by factors such as eyeglasses, contact lenses, and scars from surgeries, limits how the position of gaze can be used as a reliable indicator of human intent in assistive technologies.

Non-invasive EEG-based brain interfaces, especially designs that exploit visually evoked potentials (VEPs) have attracted increasing attention as an alternative physiological input modality (considering the fact that many neuromotor disabilities also lead to poor gaze control and visual acuity). The visual cortex response to flashing lights manifests in EEG as signals with relatively large amplitudes, and with periodic flashes, large-amplitude waves called steady state VEPs (SSVEPs) can be evoked. E. Sutter proposed using pseudorandom on/off sequences to flash multiple stimuli to induce visual cortex activity that generates EEG to track the location of gaze with high accuracy [1,2]. Bin & Gao also took a similar approach in using c-VEP to build a BCI system [3]. With the advancements in the technology, EEG acquisition systems are becoming more affordable and practical. The setup time is decreasing by using active electrodes instead of the passive electrodes and while still quite new, soon dry electrodes would decrease the setup time even more. Although these systems are still in the research settings, EEG-based brain interfaces are becoming more attractive and capable.

There has been a debate, that if the individual can control their eyes, would using an eye tracker be cheaper, less irritating and easier compared to an EEG-based brain interface. For individuals without gaze control eye trackers are of no use. It has been shown that it is possible to shift the visual attention without shifting the gaze [4,5,6,7]. This phenomenon is called covert attention. SSVEP responses under covert condition have been studied and the separability has been detected [8,9,10]. Although, in such conditions a performance decrease is expected. Furthermore, separation of overlapping SSVEP stimuli is also shown to be possible [11], which could also be used to overcome the lack of eye gaze control. A study by Golla on the individuals with cerebellar disorders [7], unveiled that the contributions of the cerebellum to attention are confined to overt manifestations based on goal-directed eye movements. They found that saccadic dysmetria did not predict performance in the covert attention paradigm. These findings suggest that brain interfaces which only utilize shifts in visual attention could be an attractive alternative to gaze control devices [12,13].

Generally speaking, even among individuals capable of controlling their gaze, both input modalities have their drawbacks. Eye trackers might not be very responsive for individuals with corrected vision, especially individuals wearing eye glasses. Working based on image analysis and dealing with rapid eye movements, eye trackers usually require expensive video capture and processing equipment [14,15]. Non-invasive VEP-based brain interfaces using visual flickering stimuli could cause irritation and fatigue and require setting up electrodes. Considering the low signal to noise ratio of EEG signals, these systems are also sensitive to environmental noise and equipment cost although decreasing is still on high. Additionally, combining the two input modalities to overcome the shortcomings of each in a hybrid BCI system has gained interest and shown to have the potential to increase the overall performance [16,17].

This study compares the two input modalities. In addition to communication efficacy, we measure user comfort and setup considerations, both of which are essential considerations when a user selects a device. A few comparisons between different brain responses and eye tracking have been done in the past. These studies were more focused on the P300 and SSVEP responses [18,19,20]. Factors such as system reaction time can affect the user experience and preferences. In this study, we utilize one of our applications, FlashPlay^TM^, using c-VEP-based EEG stimulation and eye tracking as input modalities [21]. The system can provide more applications such as typing, navigation and object manipulation with the same interface, for this study, we have chosen FlashPlay^TM^, a simple virtual game, to decrease the setup time [22].

## 2. User Interface & Application

**Figure 1:**
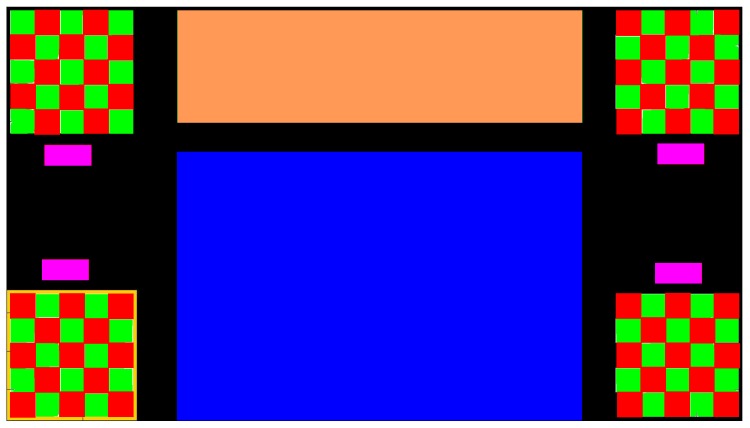
An example of the screen with four stimuli. The blue rectangle in the center shows the area reserved for visualizing the applications. The orange rectangle shows the area reserved to provide the visual feedback. The purple rectangles show the area in which the stimuli labels appear. The yellow frame around the lower left checkerboard is the indication of that checkerboard being the target in this screen shot.

We describe the user interface, which can be considered in three parts: *Stimuli*, *Application* and *Feedback* (see Figure 1). The checkerboard corners serve as the stimuli to provide the flickering pattern, alternating red and green in time for c-VEP input with no flickering for eye tracker input. Their position is chosen to provide maximum distance between the visual stimuli to mitigate inter stimuli interference. The area in the center of the screen is reserved for visualizing the *Application* and the *Feedback* combined or separated where applicable.

The system provides two separate feedbacks. First is the immediate feedback, provided by a yellow colored frame around the selected or targeted stimulus. Second is the application specific feedback. In some applications, i.e., the Maze game, this feedback is provided by an action performed by the indicator icon in response to the selected stimulus. In other applications, such as FlashType™ [23], this feedback is provided by a frame around the selected icon, symbol or text and shown in the *Feedback* section by printing the chosen target.

One of the main features of this interface is its adaptability to different applications and input modalities. Having accurate, fast and reliable responses to the specific visual stimuli, the system is highly adaptable and can be used in different applications. This adaptability is achieved by utilizing the provided stimuli in different roles. Stimuli roles are presented by stimuli labels shown beneath or above each stimulus.

## 3. Input Modalities

Both gaze tracking and c-VEP work to estimate the focal point of one’s eyes. Gaze tracking estimates directly via infrared images of the eyes themselves. Alternatively, c-VEP utilizes a unique visual stimulation per some fixed set of locations. Gaze is estimated by examining the visual cortex’s frequency response to estimate the visual stimuli which the user is looking at. The system deduces the user’s gaze point by utilizing a fixed mapping from stimuli to gaze positions.

We have made every effort possible adjusting the settings such that they are reasonable and fair for both input modalities to be able to make a just comparison. These settings include the *decision rate*, the *decision show time* between the consecutive decisions, and the area assigned to each stimulus. Decision rate has been set to 1 Hz. The *decision show time*, set to two seconds, is the duration the application will pause after executing a decision and before starting the stimulation for the next decision. This parameter can be changed based on the user preference, we have found that shorter durations increase the cognitive load on the participants, especially for the novice users. The stimuli area and location are kept the same for both input modalities. Under this design, both input modalities are bounded to the same theoretical ITR, however, as stated by Yuan [24,25], ITR is not an accurate measure of online BCI or assistive technologies due to assumptions such as equal decision probability for all the options, fixed decision rate and uniform error probability.

From the user perspective, there are two main differences between the stimuli for the two input modalities. Firstly, stimuli areas are presented by checkerboards for both input modalities, but, due ot tiring and irritating flickering effects, flickering is disabled when the eye tracker is used as the input modality. Secondly, when the eye tracker is used as the input modality, a blue circular frame presenting the estimated gaze point, is presented to the user. Currently with this BCI system estimating user gaze point using EEG in real-time is not possible, but, the eye tracker can provide gaze point estimates in almost real-time. The gaze point presentation, provides the user with a feedback. In this study, aiming for more accurate eye tracker calibrations with the head fixed eye tracker, we allowed users to make slight head movements, adjusting their gaze point to the presented estimated gaze point. While this plays an advantage towards the eye tracker and might not be possible for the individuals with severe disabilities, we found not allowing this very frustrating for the participants.

### 3.1. Code Visually Evoked Potentials

**Figure 2:**
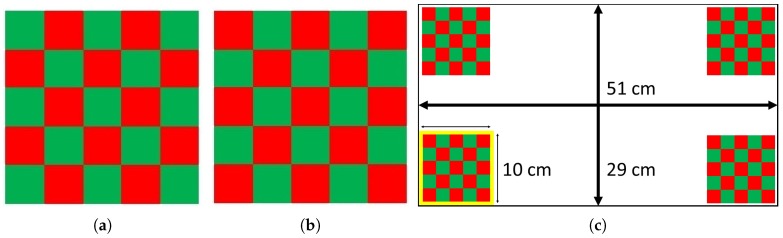
(**a**) The checkerboard pattern corresponding to the *Off* state, bit “0” in the control bit sequence; (**b**) The checkerboard pattern corresponding to the *On* state, bit “1” in the control bit sequence, and (**c**) The target arrangement of the stimuli [29].

Visual Evoked Potentials (VEPs) are the responses of the visual cortex to a flash of light. Historically, flashing at some regular frequency (i.e., Steady State Visually Evoked Potential) has been used as it provides robust signal generation and relatively simple EEG classification schemes. Code Visually Evoked Potentials (c-VEPs) are an alternate VEP which seek to improve performance beyond SSVEPs [26]. Instead of a consistent frequency, a pseudorandom binary sequence of *On*/*Off* is used for user stimulation. The *On* and *Off* states are defined based on the stimulation pattern used. For simple symbol/LED, it is just turning the symbol/LED on and off, while for a reversed color checkerboard pattern, one pattern is assigned to the *On* state and the reverse pattern is assigned to the *Off* state. Figure 2, shows the stimulus pattern for the *On* and *Off* states as well as the stimuli arrangement on the screen. Here we use a c-VEP-based stimulation method in which m-sequences [27], a special category of pseudorandom binary sequences, are used as the control bit sequences. Each stimulus is assigned a unique m-sequence of length 63 bits to control its flashing pattern. These sequences have maximum Hamming distance with their lags and their pairwise distance is large. Bit presentation rate of 110 Hz was used to present the stimuli. This combination of the control bit sequence length and bit presentation rate results in trails of 0.57 s long. A trial is a complete presentation of the corresponding patterns in the control bit sequence. For such stimuli, different parameters are important, such as the bit presentation rate, control bit sequence length, stimulus size, color, placement and the size of the blocks used in each checkerboard. The parameters in this study are set based on the previous studies [26,28].

In this study, every stimulus consists of a 5 × 5 square checkerboard and its reversed pattern. The size of every stimulus is chosen as 1/3 of the screen height. Having a wide screen layout, the specific size used, provides a well-sized stimulus, keeping the inter-stimulus distance equal to at least one stimulus. In addition, each block being 1∼2 degrees of visual field usually provides a strong response [30]. Blocks in each checkerboard are flipping their colors between *Red* and *Green* to provide stronger responses and less irritation simultaneously, the specific choice of color pairs was made based on the results of a previous study [29]. The same study shows that the black and white color pair would provide nearly similar results, useful for individuals who might be colorblind. Flipping between reversed color checkerboards provides a constant illumination, in addition every block in the checkerboard is always visible resulting in stimulation for both *On* and *Off* states. Additionally, m-sequences having an almost equal number of ones and zeros play a significant role in balancing the stimuli illumination at each time instance. The number of ones and zeros in any m-sequence is only different by one, meaning that using an m-sequence for stimulation results in showing the two patterns almost equal number of times per trial, in case of 63 bit long m-sequences the ratio of the number of zeros over the number of ones is 0.96875.

A g.USBAMP bio-signal amplifier, g.GammaBox and a single g.Butterfly electrode from g.Tec (Graz, Austria) is used for EEG data acquisition [31]. The sampling rate of 256 Hz and a single active electrode placed on top of the occipital lobe, Oz, according to the 10–20 standard has been used. A notch filter placed at 60 Hz was used to eliminate the AC power line noise. EEG signal was then filtered using a band-pass FIR filter (2.5 Hz–100 Hz). Use of a single channel has been shown to be adequate to provide highly accurate decisions and ease of use at the same time [26,28].

Definitions below are used to explain c-VEP stimuli.

- Trial, is the presentation of all the patterns corresponding to one period of the designated control bit sequence for each stimulus.
- Epoch, consists of one or several trials.

The developed c-VEP interface presents all the stimuli to the user simultaneously. Control bit sequences corresponding to every stimulus have the same number of bits. Using a common bit presentation rate, this results in trials of the same length for all the stimuli. The system presents the stimuli trials time-locked to each other so that they start and end at the same time. The onset of the trials is marked on the EEG signals using a hardware trigger provided using the parallel port and the digital input channel on the amplifier. Participants are required to behave consistently while using the system. In other words, since the system uses the gathered data from a user during a *Calibration* session to classify user intent, it is important that the users continue the same strategy they were following during the *Calibration* while using the system to do different tasks. This consistency plays a key role in keeping a high performance.

During every epoch, the stimuli will present one or more trials. Once the classifier reaches the predefined confidence threshold, stimulation stops and the system reacts to the detected command. Once the action corresponding to the detected command is complete, the user is given a short period of time to decide on the next command and the stimulation starts again. The presentation of different trials in an epoch is not distinguishable from the user perspective. In other words, the user experiences a series of different checkerboard patterns presented until a decision is made presented and presented on the screen.

### 3.2. Eye Tracking

**Figure 3:**
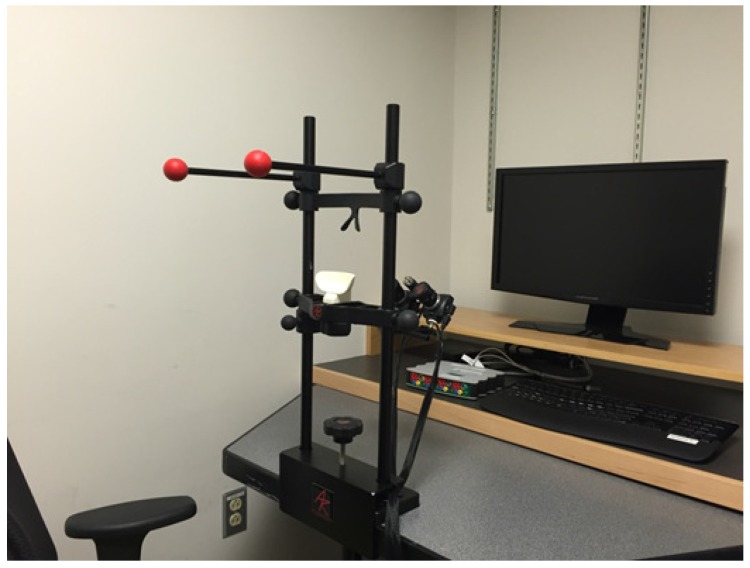
Experiment setup showing the head-fixed binocular eye tracker.

An *Arrington Research* USB220 binocular head fixed eye tracker with *ViewPoint EyeTracker* software, from *Arrington Research*, Scottsdale, AZ, USA, has been used [32]. The sampling rate of the eye tracker is 220 Hz on average. Eye tracking is done on both eyes using two independent cameras pointing at the user’s eye from approximately 7 cm. Working based on image processing principles, the sampling rate is affected by a few factors such as the processing speed and the processing load on the system, hence, it is not constant. Figure 3 illustrates the eye tracker and display assembly.

Using eye tracking as input modality is a two-step process. The first step is to find the gaze point accurately, and the second step is to determine if the user has an intent over that point. Finding the gaze point can be done by several methods [33], some of which are proprietary to the producer company. In this study, the ViewPoint EyeTracker software from Arrington Research with a USB220 Binocular eye tracker provides the gaze point estimates.

## 4. Calibration

*Calibration* is a session during which, labeled data is gathered from the user for the corresponding input modality. Depending on the stability and consistency of the *Calibration* data, there might be a need to repeat the *Calibration* session when the previously collected *Calibration* data is not performing well enough. However, the stability of the *Calibration* data depends on the input modality and might also differ from one individual to the other. In this study, *Calibration* sessions for eye tracker and the EEG-based input modalities are collected separately.

### 4.1. c-VEP

The *Calibration* for c-VEP input modality requires collecting enough responses for every stimulus to build a template response. The designed *Calibration*, presents all the stimuli simultaneously to the user. Twenty epochs are presented to the user in each *Calibration* session. While the order of the epochs is chosen randomly at run-time, 5 epochs are presented for each stimulus. Each epoch consists of 12 trials during the *Calibration* session. Discarding the first trial to decrease the visual cue effects, the above scheme results in 50 sample trial responses to each stimulus [26]. Taking the median over the time samples gathered for the trials of each stimulus results in a robust reliable template response [34].

The *Calibration* data collected for the c-VEP-based input modality is fairly stable and consistent for every participant. Although, different strategies can be used initially, to optimize the generated responses of the visual cortex for each participant, once a good *Calibration* data is collected it can be used for an extended amount of time, months for example. The main difference between different strategies is the point of focus on the stimulus. Some examples are, the center or the inner or the outer block on the diagonal pointing towards the center of the screen on every stimulus. In addition, for some users looking at the edge of the block produces stronger responses compared to looking at the center of the same block. The quality of the *Calibration* data depends mainly on factors such as electrode placement and user behavior. As long as these factors are kept fairly constant, the same *Calibration* data can be used. A more controlled study is required to estimate the overall statistics, we have been able to successfully use a *Calibration* data for a participant after a period of 12 months.

### 4.2. Eye Tracker

Calibrating the eye tracker starts with adjusting participant’s head in the head fixed setup using the chin holder, the nose bracket, and the side holders. Next, is setting up the cameras and their corresponding infrared LEDs to point at the participant’s eyes. The process continuities with adjusting the camera lens opening and focus for each eye and some software settings, such as the distance between the participant’s eyes and the screen and the eye frame placed around the eye image coming from the cameras. Once all the hardware and software settings are adjusted, it is the participant’s turn to follow a set of calibration points on the screen. The number of calibration points is adjustable. For this study, we have used 9 calibration points distributed in a 3 × 3 matrix covering all the corners and the center points on the screen. Using more calibration points usually provide more accurate calibration, but, it also requires longer calibration time. Nine calibration points usually provide a good balance between accuracy and calibration time. After the calibration, a collection of estimated gaze points is provided to the operator, which can describe how well the points are estimated. For example, after performing the calibration on a rectangular screen, the estimated gaze points should also present a rectangle with the same side ratios. If estimated *Calibration* gaze points are not representing the screen shape accurately, the calibration process should be repeated for all the points or for selected points for whom the calibration is not accurate. Next, the participant is asked by the operator to look at the four corners of the screen to make sure the estimated gaze points presented by ViewPoint software are matching closely to the actual participant gaze point.

## 5. Classification

Different classification methods have been used to classify user intent based on the input modalities.

### 5.1. c-VEP

User intent classification is done using a two-fold classifier. First using leave one out on the responses collected during the *Calibration* session, templates are built and correlation scores are obtained for every sample response in the *Calibration* data. Templates for each stimulus are built by taking the sample median among the time locked samples of trials with the same target. Use of median as the averaging method, makes the estimation up to 50% more robust to the outliers. [34]

(1) $$r_{i}^{c}=tr\left[x_{t}^{c}\right]t_{i}^{c}$$

Here, $r_{i}^{c}$ is the correlation score for channel *c* and the $i^{th}$ stimulus. $x_{t}^{c}$ represents the EEG response of channel *c* during trial *t*, $t_{i}^{c}$ represents the template response corresponding to the $i^{th}$ stimulus and channel *c* and tr[.] represents the transpose operation.

Although, in the *Calibration* data, every trail has a single target stimulus, but, the correlation scores with the other stimuli are also considered. This consideration, models the inter-stimuli interference from the non-target stimuli and makes the classification more robust. In this study, having four stimuli results in 1 × 4 correlation score vectors. Next, considering EEG as a Gaussian process and using the correlation scores, class conditional independent multivariate Gaussian densities, $P(X_{t}|s_{e_{t}}=s)$, are estimated for the responses to each stimulus. *s* represents the stimulus and $s_{e_{t}}$ represents the stimulus for epoch *e* and trial *t*. The main goal is to estimate the conditional probability of each stimulus given the EEG evidence, $P(s_{e_{t}}=s|x)$. Although it is not possible to calculate the probability of the EEG evidence, but, applying the Bayes’ rule, we can estimate the class conditional probabilities and maintain the same ratio.

(2) $$\begin{array}{cc} P(s_{e_{t}}=s|X_{t}) & =\frac{P\left(X_{t}|s_{e_{t}}=s\right)P\left(s_{e_{t}}=s\right)}{P(X_{t})} \end{array}$$

where $X_{t}=\left[x_{t}^{1},\cdots ,x_{t}^{N_{c}}\right]$. Denominator is the only term in Equation (2) that cannot be estimated, however, being a common term and not depending on the stimulus there is no need for its value. Assuming EEG evidences from different trial are independent, we can calculate the conditional probability of multiple trials in an epoch as

(3) $$\begin{array}{c} P\left(X_{e}|s_{e}=s\right)=\prod_{t=1}^{N_{t}}P(X_{t}|s_{t}=s) \end{array}$$

where $N_{t}$ is the number of trials in epoch *e*. Having 50 samples per stimulus and using only one EEG channel, there exist enough samples to use the maximum likelihood estimates for the mean and the covariance of the above densities [35]. There might be a need to collect more trials per stimulus if using more channels is desired. Collecting more trials translates to longer *Calibration* sessions which is not desirable and might not be feasible. However, introducing some structure on the covariance matrix using methods such as Graphical lasso [36] or using regularization and shrinkage methods such as Regularized Discriminant Analysis (RDA) [37] may reduce the requirement on the number of training trials.

Ultimately, we can also consider the information extracted from the context as an independent source of information and fuse this information with the EEG evidence.

(4) $$\begin{array}{cc} P(s_{e}=s|X_{e},\omega_{e}) & =\frac{P\left(X_{e},\omega_{e}|s_{e}=s\right)P\left(s_{e}=s\right)}{P(X_{e},\omega_{e})}\\ & =\frac{P\left(X_{e}|s_{e}=s\right)P\left(s_{e}=s|\omega_{e}\right)}{P(X_{e})} \end{array}$$

here, $\omega_{e}$ represents the context information while epoch *e* has been in process. Use of the context information can boost the classification accuracy as long as the context information is in line with the user intent. It can also boost the performance by decreasing the probability of the options which are determined unlikely or infeasible based on the context information. In the absence of the context information, all the options are simply considered equally probable.

Finally, a maximum a posteriori decision role is employed to choose the stimulus with the highest posterior probability as the intended stimulus of the epoch.

### 5.2. Eye Tracking

Several methods can be used for intent detection such as blinking and fixating. Targeting a group of disabled individuals, intentional blinking would not be a feasible option, leaving the fixation as a better candidate. In this study, two simultaneous methods have been used, fixation detection by ViewPoint software, and the effective position of the gaze during the decision period. A natural way of using an eye tracker is very similar to using a mouse. Gaze point will be used as the location of the mouse indicator on the screen and the intent detection will act similar to making a left click with the mouse.

The same four square areas at the four corners of the screen are used as eye tracking stimuli. To select a stimulus the user has to keep their gaze inside the area assigned to their target stimulus.

Two simultaneous methods have been used to identify the user intent, detecting a fixation point and the effective position of the gaze during the decision period.

#### 5.2.1. Fixation Based

Fixation is defined as maintaining the gaze at a specific location. A common method to detect fixation is to monitor the gaze point using a sliding time window. If the gaze point is kept in the predefined area without rapid movements, then, a fixation point can be estimated. However, parameters such as the area of the specific location, variations of the gaze point and the duration of time that the gaze has to be kept constant have a large impact on the fixation estimation quality. ViewPoint calculates fixation as the length of time that the velocity stays below the saccade velocity criterion. Fixation events are detected using the ViewPoint software with a dwell time of one second to match the system parameters and decrease the number of false positives.

#### 5.2.2. Gaze Based

The position of the gaze changes both rapidly and frequently, especially when an individual is searching over an area in the visual field. On the other hand, fixating over a point is tiring and sometimes not feasible when eye movements are not all intentional. Here we propose an intent detection method solely based on the gaze points during the decision time window. In this method, wide areas are assigned to the stimuli and only gaze locations are considered, relaxing the fixating parameters to make things easier for the user. With the decision rate of 1 Hz, the location of the gaze is monitored over the 1 s period. To estimate a single point as the intended point for each decision period, there is a need to combine the information gathered from all the gaze point estimates during that period. Here, the number of samples is about 220 samples per decision. Considering the fast and sometimes unintentional movements of the eyes, a method, robust to the outliers, would make a better estimate. Hence, we use the median as a dimensionality reduction tool which has a better tolerance to outliers. Every gaze point is defined by two values representing the horizontal and the vertical location. We estimate the intended point during each epoch by taking the median over the horizontal and vertical dimensions independently.

When a single point is estimated as the gaze point, it will be matched against the areas assigned to the stimuli. In case of an overlap between one of the stimuli and the estimated gaze point, that stimulus will be selected, otherwise, a new trial will begin.

## 6. Experiments

Ten healthy individuals, 6 females and 4 males, with normal or corrected vision between the ages of 23 and 30 were consented and participated. Participants were graduate students who were not a member of this project and nor under the influence of any chemicals, such as caffeine. Data collections were performed based on an approved IRB protocol at Northeastern University. Participants were seated comfortably in front of an LCD screen at 80 cm from the screen with their head fixed by use of the head-fixed setup. EEG was recorded using at 256 Hz sampling rate from a single EEG electrode placed at Oz positioned according to the International 10/20 system was used to record brain activity using a g.GAMMAcap (electrode cap). This electrode was grounded with respect to FPz and referenced against an ear-clip electrode placed on the right earlobe.

The experiment consists of two main parts, in which, the input modalities are different. During the first half, c-VEP was used as the input modality and during the second half eye tracker. The order of the input modalities for different participants was not counterbalanced mainly due to the limited number of participants within each vision status category. However, a long enough rest period of five minutes or more if desired by the participant, based on user judgments, were considered to eliminate the fatigue effects. In each part, participants were asked to complete four different mazes. The mazes were designed randomly beforehand and chosen to have almost the same number of decisions per stimulus. Maze 1 and 4 require 22 and maze 2 and 3 require 21 consecutive correct decisions to reach the final point. Table 1 is presenting the detailed number of consecutive correct decisions required to successfully complete each maze.

Experiments were designed to be extremely similar with input modality being the only difference.

Participants used the c-VEP-based input modality first and then used the eye tracking to perform the exact same tasks. Based on the stability of the *Calibration*, the calibration process had to be repeated a few times. For eye tracking, this was determined by looking at the estimated gaze locations which should illustrate a matrix with the same size ratio as the screen with proportional gaze points used for calibration. For c-VEP a minimum calibration accuracy of 85% was considered. For the c-VEP-based input modality, different strategies were used to achieve a *Calibration* data with good performance. Once a well-performing *Calibration* data was achieved, it was used throughout the c-VEP tasks. For eye tracking, *Calibration* was evaluated before every task and repeated if determined necessary.

Tasks were the same for both input modalities, having gone through them during the c-VEP part, participants were more familiar with the tasks during the eye tracking part. While the c-VEP input modality can incorporate the information about the context to boost the performance and the accuracy of the decisions, in this study, we have not used the context information to keep the comparison fair.

**Figure 4:**
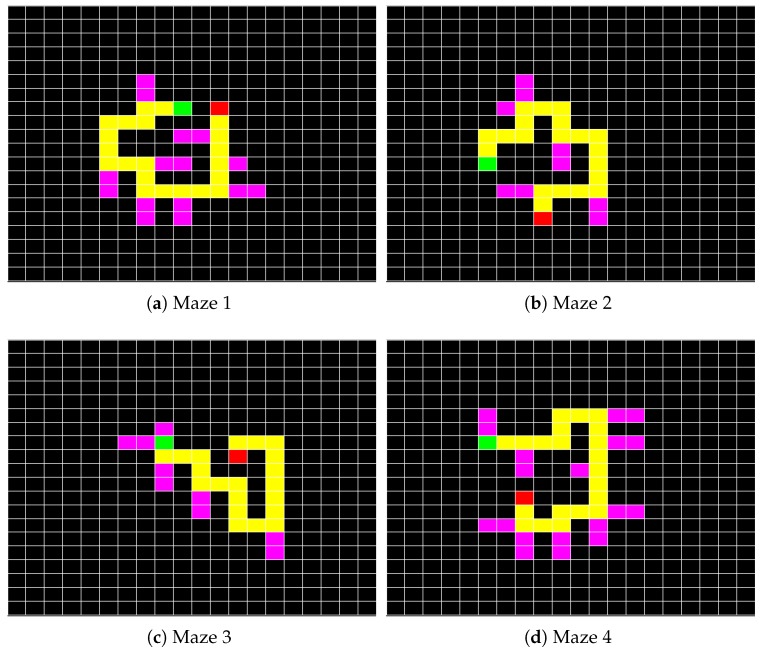
(**a**) First task; (**b**) Second task; (**c**) Third task; and (**d**) Fourth task.

Figure 4 illustrates four mazes that were presented to the participants. Mazes were presented to the participants in the same order when using different input modalities. In each maze, the start point is marked with a red block and the end point is marked with a green block. The correct path is unique, 20 blocks long and highlighted in yellow. There are incorrect, dead-end paths randomly placed along the way and highlighted in pink. The task is to move a mice indicator from the start point to get a piece of cheese placed at the endpoint. If the participant makes a mistake and leads the mice into an incorrect path, the mistake has to be corrected by coming back to the correct path.

**Figure 5:**
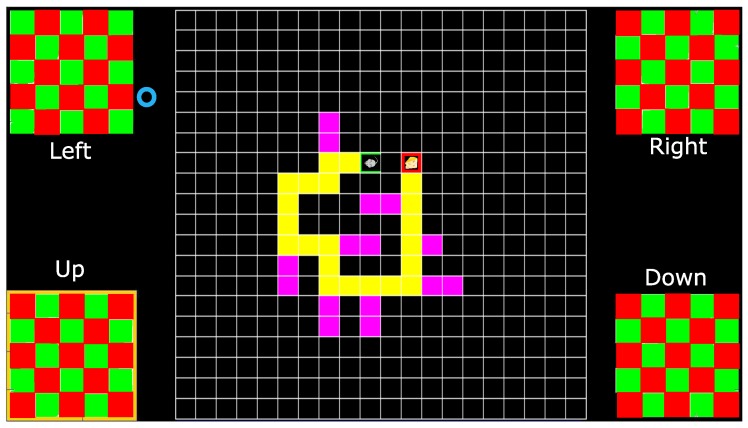
Illustration of how the screen looks like during task Maze 1. The blue circle, in the upper left corner, is an indication of the estimated gaze point presented only when eye tracking input modality is active.

Stimuli roles for this task are, turn *left*, turn *right*, go *down* and go *up* starting from the top left corner and moving clockwise. The specific roles of the stimuli in this application might suggest using the corresponding locations for the stimuli, however, to take advantage of the screen real estate, keep the application area larger and maximize the space between the stimuli, four corners where used. Figure 5 illustrates the screen during maze tasks. In this figure, the blue circle is only present when eye tracker is used as the input modality and indicates the estimated gaze point. A total timeout of 10 min is considered for each task.

## 7. Results and Discussion

### 7.1. User Experience

Participants sat in front of a 22-inch computer display at a distance of 80 cm. The height of the chair, head-fixed setup, and the computer display were adjusted such that looking straight, participant’s eyes were pointing at the top 1/3 of the computer display. After the experimentation session, every participant was asked to fill out a survey with a few questions about their preferred input modality and their reasoning. Questions and results are summarized below. Although the participants in this study were all healthy individuals with normal or corrected vision, we tried to have a variety of users covering different vision categories to evaluate both input modalities better. Factors such as wearing glasses, contact lenses or having normal vision were considered.

Among the 10 participants, 8 voted in favor of the c-VEP input modality and 2 were in favor of the eye tracking input modality. Participants were asked fill out a questionnaire after completing all the tasks which included the following questions. Q1: Which input modality you prefer? Q2: Which input modality you think was following your commands better? Q3: Which input modality you found faster? Q4: Which input modality was easier to use? Table 2 summarizes participants’ responses and vision status. Participants particularly were not comfortable with the head fixed setup for the eye tracker. In this table, we also provided their *Calibration* accuracy for the c-VEP input modality.

### 7.2. Performance Metrics

**Figure 6:**
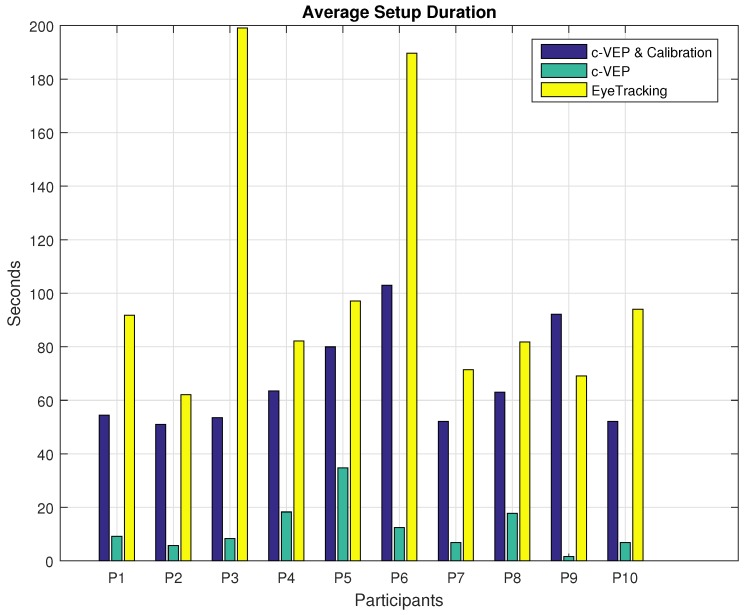
Average setup and calibration duration for each input modality. c-VEP average setup durations are reported including and excluding the one-time calibration duration.

All participants except the sixth participant were able to finish all the four tasks using both input modalities. Our sixth participant, couldn’t finish maze number 2 using c-VEP input modality and made 30% progress. The same participant had an even harder time using the eye tracker. This participant could not finish mazes 1 and 3 using the eye tracking input modality and made 0% and 50% progress respectively. Having the same decision rate of 1 Hz under the presented stimuli with four options, c-VEP and eye tracking input modalities provide similar theoretical ITR rate of 90.12 and 86.44 bits/min respectively calculated based on their average command accuracy and the ITR definition in [38]. Proceeding with different performance metrics calculated from the online experiments, first, we start with the duration of setup and calibration. Due to the sensitivity of the eye tracker, despite the efforts to use the calibration data saved after the first *Calibration*, there was a need to make adjustments before every task. These adjustments varied from one participant to the other and from recalibrating select points or all, hence, the difference in the setup time. However, except for participants P6 and P9, the same *Calibration* data was used for the c-VEP input modality for all the tasks. These two participants needed a second *Calibration* mostly due to the changes in their behavior. Duration of a single *calibration* routine is shorter for the eye tracking system, about 60 s compared to 181 s for the c-VEP system, however, the total time spent calibrating the system is the main concern. Table 3 illustrates the total time spent on setting up and calibrating each input modality for the session. More insight can be achieved by looking at the average time spent on setup and calibration per task. Figure 6 shows the average setup and calibration duration for each input modality averaged over the four tasks performed. Since the c-VEP input modality mostly uses only a single *Calibration* data, collected over a duration of 181 s with the settings used, the average is provided including and also excluding this time duration. For the eye tracking system, given this was a fixed head setup, the setup and *calibration* were not separable in time as most of the actions were taken towards achieving a better *calibration*. Since the *calibration* for c-VEP input modality is not repeated for each task, the duration of time spent for *Calibration* becomes negligible as the number of tasks increase. Initial setup time for c-VEP input modality, considering the use of a single measurement electrode was minimal and is included in the setup time. Before every task, there has been a quick EEG signal monitoring to make sure of an acceptable electrode connection and the time duration was included in the setup time. For all the participants except P9, the total calibration time for the eye tracker has been longer than c-VEP. While the setup times excluding the c-VEP *Calibration* sessions which rarely require repetition are considerably less than eye tracking setup time. *Calibration* duration for participant number 3 and 6 are much longer compared to other participants, one observation is that these participants tend to squint their eyes, which was resulting in the eye tracking losing the lock over their pupils. Participant number 6 had a tendency to squint more to the point that it even affected her ability to complete the tasks using both input modalities. This participant was not able to finish one of the tasks using c-VEP and two of them using eye tracking input modality.

**Figure 7:**
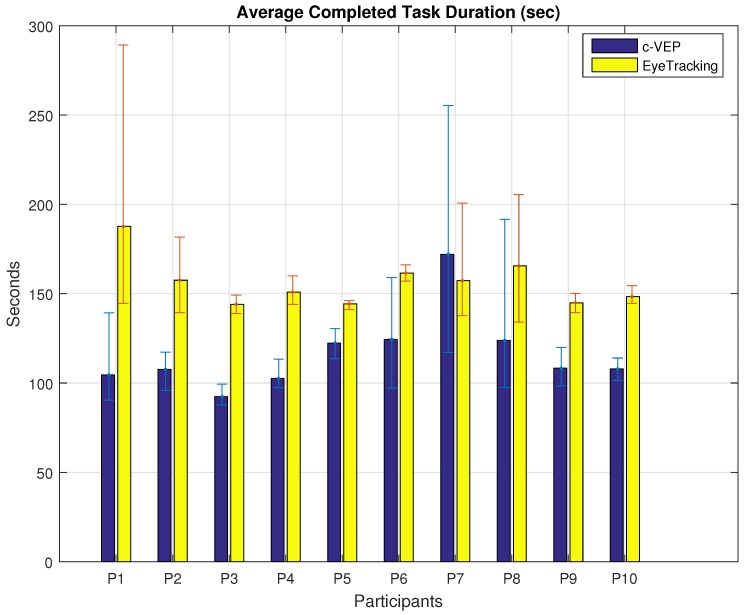
Average task duration for different input modalities. Error bars indicate the minimum and maximum duration for each participant.

Next, is the average time each participant spent to complete the tasks with the same input modality. Figure 7 shows the average time duration of the completed tasks performed with the same input modality. The results show that the average time required to complete a task using c-VEP input modality is less than eye tracking input modality except for participant P7. A paired *t*-test between participant’s matching task durations reveals a *p* value of 4.9 × 10^−7^ with mean values of 115.8 and 155.9 and standard deviations of 31.84 and 27.34 for c-VEP and eye tracking input modalities. In this calculation, only the completed tasks were considered, four tasks for each participant, except participant number 6 who had completed 3 out of 4 tasks using c-VEP and 2 out of 4 using eye tracking.

**Figure 8:**
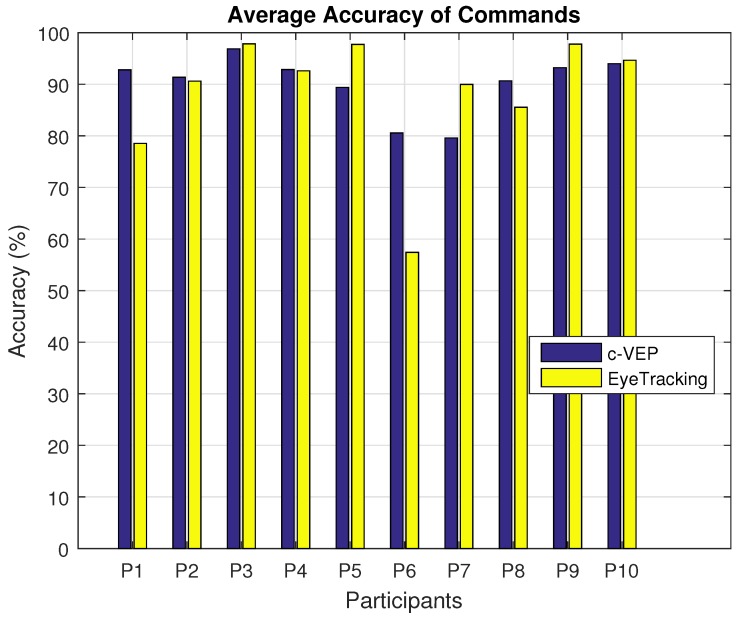
Average accuracy of the decisions made by the two input modalities. A paired *t*-test on participant’s accuracy on the same tasks reveals (*p* value <0.57) no significant difference with regards to the accuracy between c-VEP and eye tracking.

Next factor is the accuracy of the decisions made by the two input modalities. Figure 8 shows the average accuracy of the decisions made by the two different input modalities performing the four tasks for each participant. The average accuracy for the c-VEP input modality for each participant is consistently above about 80% and even most of the time above 90% Average accuracy among all participants has a mean of 92.6% and 91.4% and standard deviation of 2.74% and 7.8% for c-VEP and eye tracking input modalities respectively. For most users the accuracy of both input modalities is close, however, there are some exceptions like participants *P*1, *P*5, *P*6 and *P*7 for whom the difference is more. A paired *t*-test between participants’ accuracies on the same tasks reveals a *p* value of 0.57 with mean values of 90% and % and standard deviations of 9% and 18% for c-VEP and eye tracking input modalities respectively. This test is highly affected by the poor performance of participant 6 as indicated by the average accuracy for this participant. Excluding this participant results in a *p* value of 0.83 and standard deviations of 7.7% and 11.7% for c-VEP and eye tracking respectively. The high *p* value indicates the accuracies of the two input modalities are not significantly different, which in turn is an indication of both input modalities being viable choices. The lower standard deviation of the c-VEP input modality can be an indication of this input modality being more robust. Since all the participants except participant 6 were able to finish all of the tasks, the variability in the accuracy does not seem to limit the usability of either input modality. In cases similar to our 6th participant, changing interface parameters such as *decision rate* might be effective.

**Figure 9:**
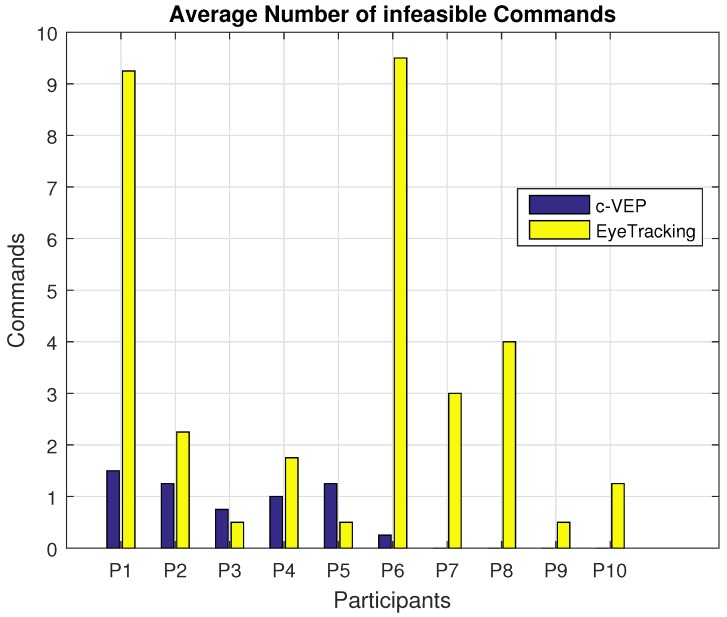
Average number of infeasible decisions made by the two input modalities.

Next, we would like to present the number of infeasible decisions made by the two input modalities. An infeasible decision is defined as a command that is not executable, i.e., when the mice indicator is facing a wall it is not possible to pass through the wall. In such situations, a command to move the mice indicator through the wall is considered as an infeasible command. For the eye tracking input modality, choosing a point out of the stimuli area is also considered as an infeasible command. A point chosen outside of the stimuli area would mean the participant has spent most of the decision time duration focusing outside of the stimuli area. Figure 9 shows the average number of infeasible decisions made by each input modality.

Between the two classification methods used for intent detection using the eye tracker, in this study fixation method was only able to detect an intent a total of seven times and the rest of the detected intents (>100 per participant) were done by the second eye tracker-based classification method. Participants, in general, had a hard time fixating on the points and even with the relaxed fixation parameters, such as drift speed and distance.

## 8. Conclusions

It is hard to find a definite answer about the best input modality due to the dependencies on many factors such as equipment and algorithms used. Here, we used the same system and the same tasks with different input modalities as the variant to be able to make a just comparison between them. Although a larger group of participants would be needed to make conclusions, our observation in this limited study shows that even healthy individuals, who have complete control over their body and more specifically eyes, might prefer c-VEP over eye tracking. This is considering the fact that the participants were given the freedom of making slight adjustments to their position to match the estimated gaze point with their actual gaze point during the eye tracking calibration routine. In this study, we tried to keep the application and most of the settings the same to make the experiments differ just in the input modality. While one might claim that there might exist better eye tracking or EEG-based interfaces, here, we have tried to make use of a fast and reliable system to make the comparison as fair as possible. Factors such as system reaction time, the irritation from the stimuli and tiring effect of staying still were the top factors affecting participant preferences. Adjusting the eye tracker for individuals wearing eyeglasses was especially very hard. Eye tracking equipment capable of tracking head movements is expected to provide ease of use and more comfort. Eye trackers geared towards being assistive devices such as Tobii-DaynaVox might provide a better performance.

Based on the observations in this study, setup and calibration durations for the c-VEP input modality are considerably less than the eye tracking. The short setup time of the c-VEP input modality partly comes from the fact that our system uses only a single EEG electrode. The *Calibration* was more stable for the c-VEP input modality, while, the eye tracking was susceptible to even slight head movements. Code-VEP input modality performed faster for most of the tasks, even though, the flickering effect made some users feel it took longer than the eye tracking tasks. Although a larger group of participants can shed more light on how the two input modalities would be compared, our results show that at least, under same decision-making time constraints both input modalities are viable choices and can be chosen based on the user preferences. Additionally, since nine out of ten participants were able to finish all the tasks using both input modalities, it seems that an average performance of 80% or higher would enable the effective use of either input modality.

## Figures and Tables

**Table 1:** Number of consecutive correct decisions required for each maze.

Maze ID	Left	Right	Up	Down	Total
1	4	6	6	6	22
2	3	6	4	8	21
3	2	6	6	7	21
4	4	6	4	8	22

**Table 2:** User response summary, B stands for Brain Interface (c-VEP), E stands for Eye Tracker. Calibration accuracy is estimated based on the *Calibration* data.

Participant ID	Vision Status	Q1	Q2	Q3	Q4	c-VEP Calibration Accuracy
P1	Corrected (CL)	B	E	B	B	98.5
P2	Normal	E	E	E	E	96.5
P3	Normal	E	E	B	B	99.5
P4	Corrected (G)	B	B	B	B	95.5
P5	Normal	B	B	B	B	91.5
P6	Normal	B	B	B	B	86.5
P7	Normal	B	E	E	E	93
P8	Corrected (G)	B	B	B	B	93.5
P9	Corrected (CL)	B	E	B	B	93.5
P10	Normal	B	E	B	B	93.5

**Table 3:** Total time spent setting up and calibrating each input modality for each participant only considering the tasks completed.

Participant ID	c-VEP (s)	Tasks Completed with c-VEP	Eye Tracker (s)	Tasks Completed with Eye Tracker
P1	218	4	366	4
P2	204	4	247	4
P3	214	4	795	4
P4	254	4	327	4
P5	320	4	387	4
P6	373	3	413	2
P7	208	4	285	4
P8	252	4	326	4
P9	369	4	275	4
P10	208	4	375	4

## Abbreviations

The following abbreviations are used in this manuscript:

EEG	electroencephalography
ITR	Information Transfer Rate
BCI	Brain Computer Interface
SSVEP	Steady State Visually Evoked Potentials
VEP	Visually Evoked Potentials
c-VEP	Code Visually Evoked Potentials
